# The 10,000 PhDs project at the University of Toronto: Using employment outcome data to inform graduate education

**DOI:** 10.1371/journal.pone.0209898

**Published:** 2019-01-16

**Authors:** Reinhart Reithmeier, Liam O’Leary, Xiaoyue Zhu, Corey Dales, Abokor Abdulkarim, Anum Aquil, Lochin Brouillard, Samantha Chang, Samantha Miller, Wenyangzi Shi, Nancy Vu, Chang Zou

**Affiliations:** 1 Department of Biochemistry, University of Toronto, Toronto, Ontario, Canada; 2 School of Graduate Studies, University of Toronto, Toronto, Ontario, Canada; Indiana University Bloomington, UNITED STATES

## Abstract

The purpose of the 10,000 PhDs Project was to determine the current (2016) employment status of the 10,886 individuals who graduated from the University of Toronto with a PhD in all disciplines from 2000–2015. Using internet searches, we found that about half (51%) of the PhD graduates are employed in the post-secondary education sector, 26% as tenure-track professors, with an additional 3% as adjunct professors and 2% as full-time teaching-stream professors. Over the time-period 2000–2015 there has been a near doubling in PhD graduates with the biggest increase in graduation numbers for the Physical (2.6–fold) and Life Sciences (2.2-fold). Increasingly, these graduates are finding employment in the private and public sectors providing the highly qualified personnel needed to drive an innovation economy.

## Introduction

### The need for PhD employment outcome data

The research enterprise within universities and affiliated institutions is driven largely by graduate students and postdoctoral fellows in an apprenticeship model where supervisors train the next generation of scientists and scholars. But, do PhD graduates necessarily follow in their supervisors’ footsteps in an era of flattened university hiring and increasingly competitive research funding? If not, then where exactly do today’s PhDs end up working?

Employment outcome data for PhD graduates is limited [1–7] making it difficult for educators to know what skills and knowledge graduates will need for career success when they complete their programs. To fill the gap, groups like the Council of Graduate Schools (CGS) have called for the systematized tracking of career pathways and enhanced professional development activities [8,9]. Indeed, a 2018 Consensus Study Report [10] of the National Academies of Science, Engineering and Medicine recommends that “Biomedical research institutions should collect, analyze, and disseminate comprehensive data on outcomes, demographics, and career aspiration of biomedical pre- and postdoctoral researchers using common standards and definitions developed by the institutions in concert with the National Institutes of Health.” In response, a consortium of nine US research institutes has launched a new initiative “The Coalition for Next Generation Life Science” (www.nglscoalition.org) focused on transparency to make “meaningful data on career outcomes available to trainees” [11].

In this paper we describe the results of 10,000 PhDs Project, which determined the current (2016) employment positions of the 10,886 individuals who graduated with a PhD in all disciplines from the University of Toronto (U of T) between 2000 to 2015 with a success rate of over 85%. An initiative of the School of Graduate Studies (SGS) at U of T, the 10,000 PhDs Project relied entirely on internet searches of publically-available data sources to track the career trajectories of graduates. No surveys were conducted and no individuals were contacted. The same methodology (described below) was used by the Higher Education Quality Council of Ontario (HEQCO) in their survey of 2009 Ontario PhD graduates [12].

Each of the five main employment sectors–post-secondary education (PSE), private, public, charitable and individual–were subdivided by job description. The data were analyzed for each of the four U of T graduate divisions: humanities, social sciences, life sciences, and physical sciences. The data were further analyzed by gender, by citizenship (Canadian/Permanent Resident/International), and by employment location.

## Methods

A complete description of the search methodology, definitions of employment sectors, job descriptions, and the data fields in the survey form are provided in Supporting Information (S1 Methodology and Survey Form). Briefly, a team of researchers was recruited from a pool of senior undergraduates and graduate students at U of T. Researchers went through an extensive training period that included pilot searches, job classifications and confidentiality. Lists of PhD graduates from existing graduate student registry data were provided by the SGS, which included the following identifiers:

■ Year of Graduation                ■Graduate Division

■ Full legal name                       ■Department/Graduate Unit

■ Field of Study                        ■Gender

■ Thesis Title                            ■Country of Citizenship

■ Supervisor(s)                        ■Status in Canada

Starting with the year of graduation researchers conducted systematic searches on Google using the full name, PhD (University of Toronto) and field of study to determine the current and any previous employment, as well as any further education pursued by the graduates. A secondary search used Google Scholar to perform a literature search of the publication history of the alumnus to determine location/institution information as well as time spent at the institution by assessing author affiliation and publication dates. A tertiary search used the supervisor or home department websites to retrieve any alumni information from these public sources. The most useful sources of information were: Google Scholar and on-line publications, university and corporate web-sites and directories, personal web-sites and Linked-In. Access to the survey interface was restricted to researchers based on their unique University of Toronto Identifier (UTORID). The data was entered into individual survey forms (See Supporting Information) created in and uploaded securely to SharePoint. The researchers who participated in data collection no longer had access to the data once each submission was completed. No data was ever stored on personal computers. The researchers worked together to do individual searches one calendar year at a time in random order, taking about 15–30 minutes per search and inputting of data. The searches were carried out over an 8-month period from June 2016 to January 2017 at a total cost of $50,000 to pay the part-time student researchers.

Once verified and entered into the survey form (Supporting Information), researchers filled in other sections that included PhD Field of Study, Research Activity, Employment Sector, and Skills pertaining to their current employment. Data was only recorded if it was found in two or more reliable internet sources such as publications, university or company web-sites and staff directories. The research coordinator (Liam O’Leary) and project supervisor (Reinhart Reithmeier) reviewed the annual data once completed to look for any anomalies or inconsistencies, especially with regard to job classification.

Using this methodology, we were able to locate 85% of the PhD graduates. These data sets are a collection of the employment status in 2016 of the PhDs who graduated from the U of T from 2000 to 2015 without any personal identification. The data for U of T, the four graduate divisions (Humanities, Social Sciences, Life Sciences and Physical Sciences), Faculties, and individual graduate departments are represented as tables and pie charts were generated using Excel spreadsheets of the exported data.

The research protocol for the 10,000 PhDs project was reviewed by the Research Oversight and Compliance Office at the University of Toronto. Because the project would only be accessing publically-available data and no individuals would be contacted or identified as part of this research project, the Office confirmed that Research Ethics Board approval would not be required. Researchers who were collecting data signed a confidentiality agreement that was approved by legal counsel at the University of Toronto.

## Results and discussion

### Increase in number of PhD graduates

The U of T is Canada’s largest university with approximately 75,000 undergraduates and 17,000 graduate students enrolled in masters, professional masters and PhD programs. U of T is the highest-ranked research-intensive university in Canada and among the top-ranked public universities in the five most prestigious international rankings: #22 in the Times Higher Education (https://www.timeshighereducation.com/student/best-universities/best-universities-world), #28 in the QS World Rankings (https://www.topuniversities.com/university-rankings), #23 in the Shanghai Ranking Consultancy (https://www.statista.com/statistics/226665/academic-ranking-of-world-universities/), #20 in the U.S. News Best Global Universities and #4 by the National Taiwan University (http://nturanking.lis.ntu.edu.tw/). Comparable public universities in the United States would include the University of California universities, University of Michigan, University of North Carolina, University of Wisconsin-Madison, Pennsylvania State University, University of Illinois, Purdue University, University of Texas, Texas A&M, University of Washington, University of Minnesota and University of Massachusetts, among others.

**Fig 1:**
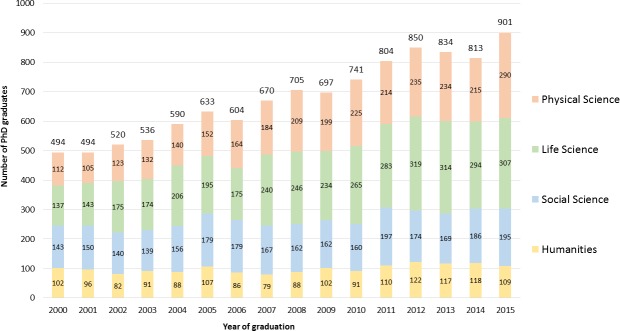
Overall number of PhD graduates from 2000 to 2015 at the University of Toronto, sub-divided by the four graduate divisions (Physical Sciences, Life Sciences, Social Sciences, Humanities).

Between 2000 and 2015, U of T witnessed a near 2-fold increase in the annual number of PhD graduates from 494 to 901, in part as government-driven strategies to invest in highly qualified personnel to build an innovation-based economy (Fig 1). The School of Graduate Studies at U of T is organized into four divisions: Physical Sciences (including the Faculty of Engineering), Life Sciences (dominated by graduate students in the Faculty of Medicine), Social Sciences (including the Ontario Institute for Studies in Education), and Humanities. The division that saw the greatest increase in the number of graduates was Physical Sciences (2.6-fold), followed by Life Sciences (2.2-fold) and then Social Sciences (1.4-fold). There was no increase in the annual number of Humanities graduates, which has remained steady at about 100 for each year.

### PhD graduates are employed in various sectors of the economy

**Fig 2:**
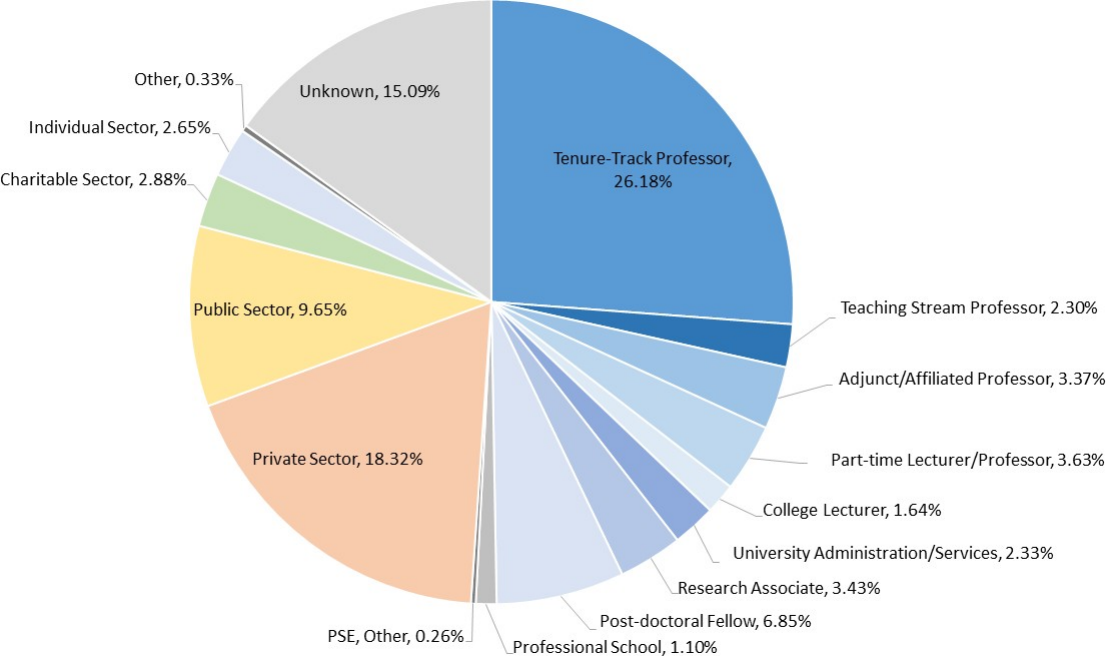
Major employment sectors of U of T PhD graduates (n = 10886) from 2000 to 2015 in different sectors determined using internet searches. The Post-Secondary Education (PSE) Sector in blue includes tenure-track professors, adjunct professors, teaching-stream professors, full-time and part-time lecturers, university administrators, research associates, and those continuing their education as post-doctoral fellows and in professional schools.

Fig 2 shows the distribution of U of T PhD graduates from 2000–2015 in various employment sectors, as determined via internet searches carried out in 2016. Using this methodology, we were able to determine the current employment positions of 85% of the graduates. Of these, about half (51%) are currently employed in the post-secondary education (PSE) sector; 26% are employed as tenure-track professors. Tenure-track professors are those who are tenured or tenure-stream, teach at the undergraduate and graduate level in their discipline and typically conduct research support by grants, supervise graduate students and post-doctoral scholars and publish in their field of study. An additional 2.3% are employed as full-time teaching-stream professors. These professors are primarily focused on teaching and pedagogy, typically employed in small undergraduate-degree granting universities or in colleges. Another significant group at 3.4% are adjunct professors who are commonly employed as independent research scientists in university-affiliated research institutes. They have a similar job description to tenure-track faculty with a primary focus on research, training graduate students and post-doctoral scholars and may be involved in teaching activities. Thus, about 1/3 of U of T PhD graduates from 2000 to 2015 currently hold full-time positions as university professors. Others are employed in the PSE sector as full-time (1.6%) or part-time/sessional lecturers (3.6%), mostly in the Humanities, and as research associates (3.4%), or university administrators (2.3%). Some, mostly recent (2012–15) graduates, are continuing their education as post-doctoral fellows (6.9%) or in professional schools (1%). 18% are employed in the Private Sector, 10% in the Public Sector, and 3% each in the Charitable/Not-for-Profit Sector and the Individual/Self-employed Sector.

The positions of 15% of graduates could not be determined by internet searches alone and they are classified as “unknowns’. For some individuals we could not find two independent sources of career information and others did not have a professional (web-site, LinkedIn, etc.) on-line presence. Some recent (2015) graduates still self-identified as PhD students. The 10,000 PhDs Project did not determine unemployment rates or salary levels. However, 2016 census data [13] from Statistics Canada showed that unemployment rate for the 176,750 PhDs in the Canadian job market, including those with PhD degrees from abroad, was 5.1%, which is lower than the 7.7% rate for the general population. A Statistics Canada study [14] of 2005 PhD graduates found that their median income was $65,000 two years after graduation, but this includes many who were employed as post-doctoral fellows and is much higher than the median income ($41,400) of Canadians employed on a full-time basis.

**Fig 3:**
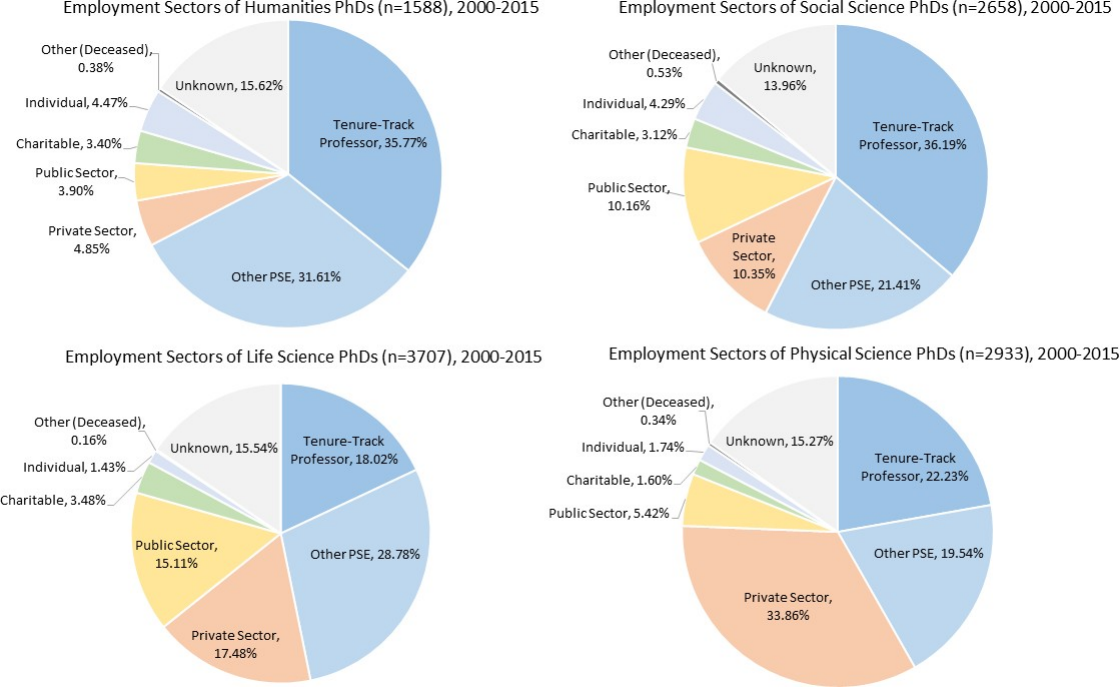
Major employment sectors of U of T PhD graduates by graduate division (Humanities, Social Sciences, Life Sciences, Physical Sciences).

The percentage of graduates currently employed as tenure-track professors varied with graduate division (Fig 3.), with the highest in Humanities (36%) and Social Sciences (36%), followed by Physical Sciences (22%) and Life Sciences (18%). In contrast, the percentage of graduates working in the Private Sector was highest for Physical Sciences (34%), followed by Life Sciences (17%), Social Sciences (10%) and Humanities (5%). By Faculty, the Rotman School of Management had the highest percentage (72%) of their PhD graduates currently employed as tenure-track professors, followed by the Factor-Inwentash School of Social Work (58%), the Lawrence S. Bloomberg Faculty of Nursing (48%) and the Faculty of Kinesiology and Physical Education (44%). In contrast, 15% of the Life Science graduates in the Faculty of Medicine are currently employed as tenure-track professors.

### Employment in the Private and Public Sectors

**Fig 4:**
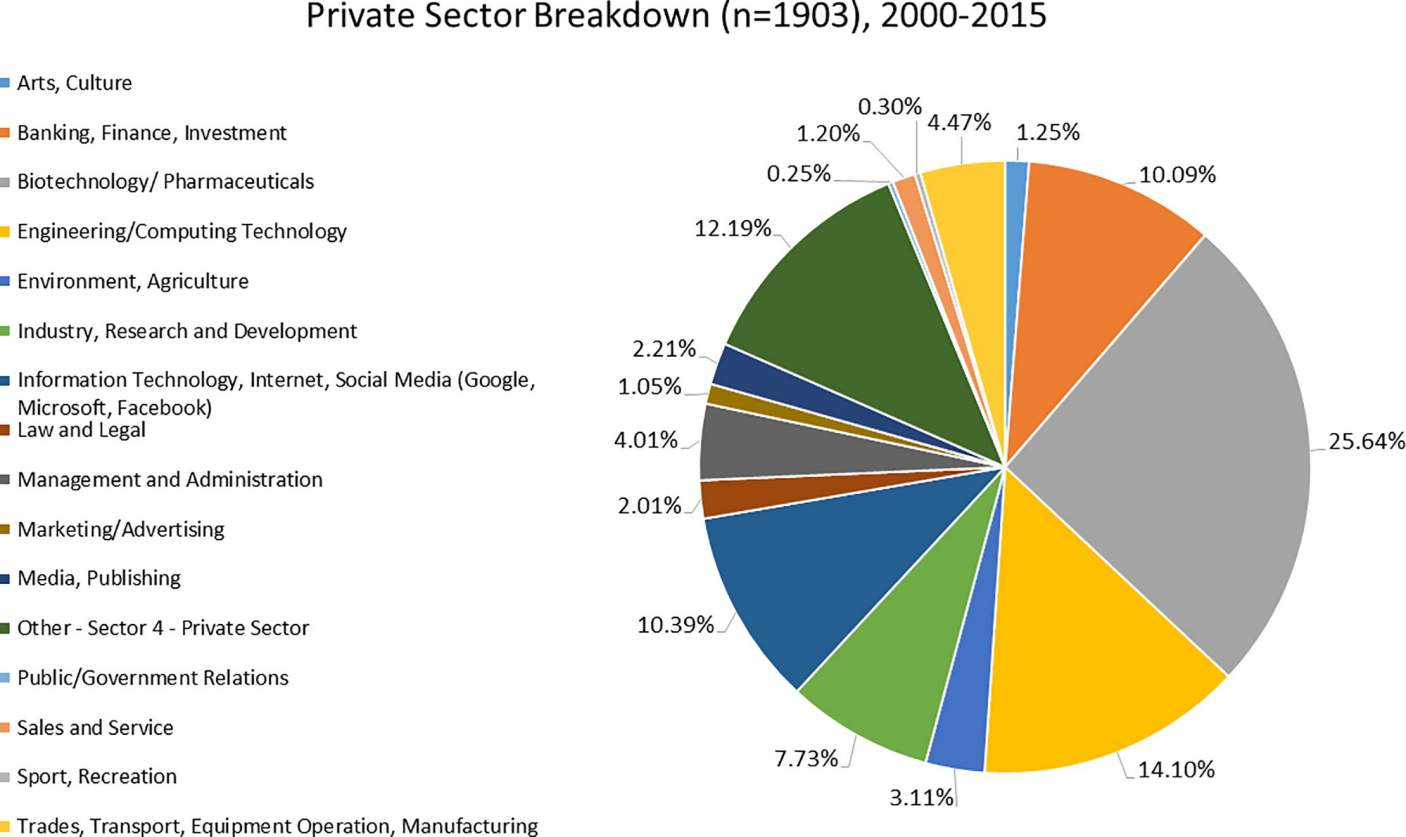
Employment of University of Toronto PhD graduates in different industries in the Private Sector (n = 1993).

Fig 4 shows the distribution of PhD graduates currently employed in different industries in the Private Sector. At 26% of the those employed in the Private Sector, Biotechnology/Pharmaceuticals employs the highest number of graduates, primarily from Life Sciences. Companies in Engineering/Computing Technology (14%), Information Technology (10%), and Banking, Finance and Investment (10%) mainly employ Physical Science graduates working for major Canadian banks and investment firms. For graduates in IT, the major employers are international companies like Google, Microsoft, Intel and IBM with graduates working in Canada and the USA. The third-ranked term “Other” refers mainly to those working in private firms as psychologists, social workers, etc. In the Public Sector, major employers of PhD graduates (mainly Social Sciences and Life Sciences graduates) are federal, provincial and municipal governments and hospitals. These individuals are commonly employed to work on research projects and in policy.

### Employment sector trends from 2000–2015

**Fig 5:**
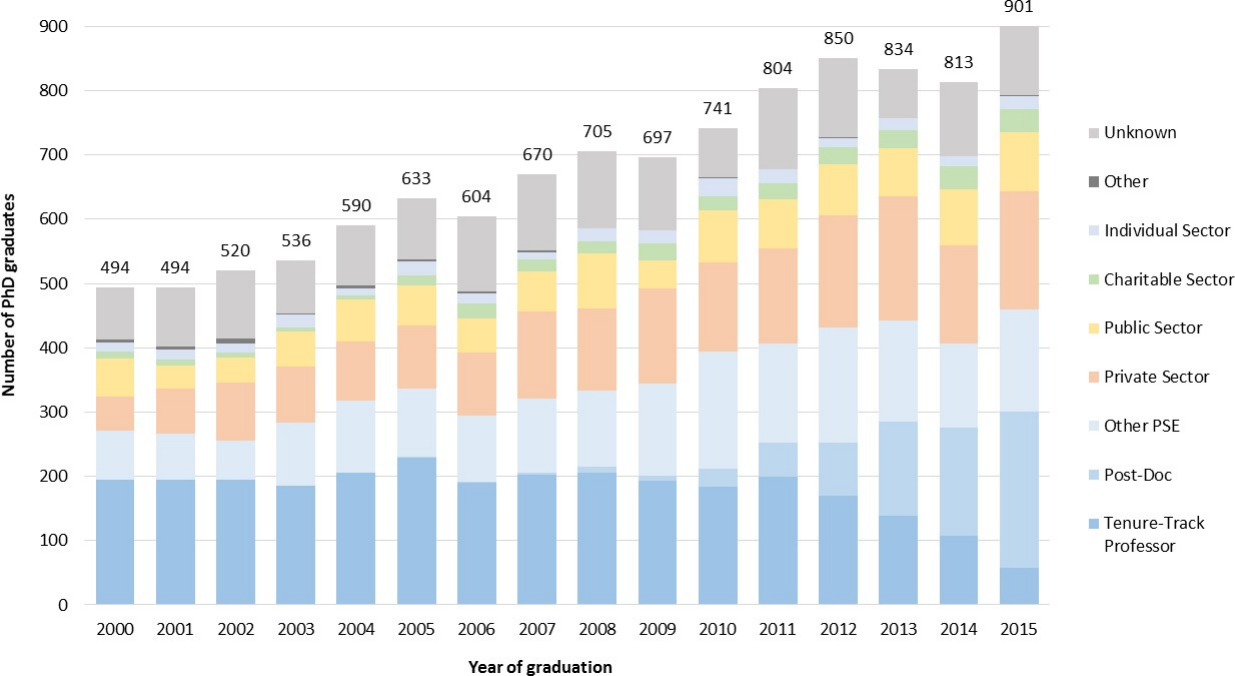
Major employment sectors of U of T PhD graduates from 2000 to 2015.

Fig 5 shows major employment sectors for each year from 2000–2015. The uptake into tenure-track positions has remained constant over the time period 2000–2011 with about 200 graduates from each year assuming tenure-track positions. While it would appear that more recent graduates are finding tenure-track in numbers lower than older cohorts, this is likely a temporary phenomenon associated with the multi-year process, including post-doctoral positions, of applying for, and finally being hired into, such positions. The tenure-track employment rates of 2012–2015 graduates would likely reach the same levels as earlier cohorts by 2020.

The vast majority of graduates currently employed as post-doctoral fellows are in the Physical and Life Sciences. Interestingly, only about ¼ of the 2015 graduates are post-doctoral fellows, with the remainder moving directly into employment. Thus, the majority of the most recent PhD graduates do not follow the traditional pathway of pursuing a post-doctoral fellow position, a necessary prerequisite for faculty positions in the Life and Physical Sciences. The number of graduates who are currently post-doctoral fellows decreased steadily for those who graduated earlier than 2015 (Fig 5) with very few 2011 graduates currently employed at post-doctoral fellows. Projecting forward from 2011, if the number of PhD graduates who become tenure-track professors remains constant at about 200 per graduating year (Fig 5, dark blue bar) about half of the ~250 individuals who graduated in 2015 who are currently in post-doctoral positions (Fig 5, light blue bar) will become professors. Given that the number of graduates who obtain tenure-track positions has remained constant and the number of graduates has almost doubled, the percentage of recent graduates who become professors is about half of the 40% of 2010 graduates.

The bar graph shows that while the number of PhD graduates employed at tenure-track professors has remained constant, the number finding employment in other sectors particularly the private and public sectors has increased. This is particularly true for Physical and Life Science graduates, who make up the bulk of the increase in number of graduates. Examples of job titles (President, Vice-President, Chief Executive Officer, Chief Innovation Officer, Director, Senior Scientist, Senior Consultant, Manager, Partner, etc.) indicate that graduates working in the Private and Public Sectors have assumed a variety of managerial or executive leadership positions.

### Where in the world are U of T PhD graduates employed?

About 2/3 (66%) of the found U of T PhD graduates are employed in Canada, 21% in the United States and 13% internationally (Table 1). The majority (76%) of Canadian graduates are employed in Canada, while 17% go to the US, often temporarily as post-doctoral fellows. 7% of Canadian graduates are employed outside of North America. Permanent residents tend to stay in Canada (56%), although about 25% go to the United States. About equal percentages of international graduates stay in Canada, go to the US or return home, although this varies with citizenship. For example, 68% of American graduates from U of T return to the US for employment, most commonly as tenure-track professors in the Humanities.

**Table 1:** Employment locations of found University of Toronto PhD graduates (2000–2015) and sorted as Canadian Citizens, Permanent Residents and International graduate students at time of graduation.

Origin	Total Found PhD Graduates	Employed in Canada	Employed in USA	Employed Outside Canada/USA
U of T	9243	6136 (66.4%)	1918 (20.8%)	1189 (12.9%)
Canadian Citizen	6256	4772 (76.3%)	1068 (17.1%)	416 (6.6%)
Permanent Resident	1929	1083 (56.1%)	488 (25.3%)	358 (18.6%)
International	1058	281 (26.6%)	362 (34.2%)	415 (39.2%)

Of the PhD graduates who are currently tenure-track professors, 60% are employed in Canada at over 60 different universities with U of T, York University and Ryerson University as the top employers–all in the Toronto area, suggesting a strong local geographic preference. Of all the professors hired at U of T over the period 2000–2015 about 15% are U of T PhD graduates; 85% of the hires are graduates from other universities. 24% of tenure-track professors are employed in the US, and 16% in international universities, mostly in China/Hong Kong, Singapore, and the Middle East.

### Gender

An about equal number of women (49%) and men (51%) graduated with a PhD from U of T from 2000 to 2015 (Table 2), although this varies by division: women make up 24% of PhD graduates in Physical Sciences, 55% in Life Sciences, 65% in Social Sciences and 55% in Humanities. Women PhD graduates are well-represented in tenure-track (46%) and full-time teaching-stream (51%) professor positions. At U of T there is a nearly equal distribution of the 257 male and female U of T PhD graduates from 2000 to 2015 currently employed as tenure-track professors, while women predominate teaching-stream professor positions.

**Table 2:** Gender distribution of University of Toronto PhD graduates (2000–2015) and those who are currently employed as tenure-track or teaching-stream professors overall, and for University of Toronto hires.

Gender	Total PhDs	Overall		University of Toronto	
		Tenure-track	Teaching-stream	Tenure-track	Teaching-stream
FemaleMale	5337 (49%) 5549 (51%)	1316 (46.2%) 1534 (53.8%)	128 (51.2%) 122 (48.8%)	130 (50.6%) 127 (49.4%)	45 (57.0%) 34 (43.0%)
Total	10886	2850	250	257	79

### Other comparable PhD outcome studies

A PhD outcome survey for 2005–13 graduates was recently completed by the University of British Columbia [1] using a combination of surveys (49% response rate) and internet searches (91% overall completion rate). The results obtained are remarkably similar to the 10,000 PhDs Project. They found that of the 3750 graduates: 51% are in the PSE sector, 26% in the private sector and 13% in the public and not-for-profit sectors. 60% are employed in Canada and again, Canadian citizens tend (75%) to be employed in Canada. About 1/3 of International student graduates stay in Canada, 1/3 go to the USA, and 1/3 return to their home countries.

An April 26, 2016 report [12] by Linda Jonker from the Higher Education Quality Council of Ontario (HEQCO) entitled “*Ontario’s PhD Graduates from 2009*: *where are they now***?”** found that 51% of PhD graduates from 2009 graduating all Ontario universities (~30% from U of T) were currently employed within the PSE sector, 29% as university professors, 4% primarily teaching at a university both full-time and part-time, 3% affiliated as status-only professors, 9% in research at universities (as post-doctoral fellows, research associates, etc.), 2% in colleges, and 3% in other roles at universities. 34% of the graduates are employed outside academia and employment information for the remaining 15% could not be found. The corresponding data from the 10,000 PhDs Project for 2009 U of T graduates is very similar with 51% in the PSE sector and 27.5% as tenure-track professors and 5% as full-time university lecturers or teaching-stream professors. Similarly, the current employment status of 15% of the 2009 U of T PhD graduates could not be determined. Both studies found that about 2/3 of PhD graduates that were found are employed in Canada. The HEQCO study used internet-based searches to identify where PhD graduates are currently working and the 10,000 PhDs Project used the same methodology. The finding that the percentage of U of T PhD graduates who are currently tenure-track professors is similar to the percentage of PhD 2009 graduates from all Ontario universities stands in contrast to the United States where institutional prestige plays an enormous role in shaping faculty hiring across disciplines [15]. An earlier 2015 Conference Board of Canada survey [2] found that about 40% of all PhDs working in Canada were employed in the PSE sector, 18.6% as professors, but these numbers include individuals with PhDs from universities outside Canada.

Using internet searches, Stanford University [4] determined the initial employment (within 1 year of graduation) and current (2013) employment of two graduation cohorts: a 10-year cohort (2002–03 graduates) and a 5-year cohort (2007–09). They located 2,420 graduates and determined 74% of the initial and 81% of the current positions. In terms of current employment, 45% are in the academic sector, 32% in business, 2% in government and 3% in the non-profit sector. Stanford was the top employer of Stanford graduates as tenure-track professors. PhD alumni in Humanities have a high percentage (77%) of being currently employed in the academia while engineering graduates (48%) tended to be employed in business most commonly by companies like Google and Intel. There is a strong local geographical preference for the San Francisco Bay area for Stanford graduates as we found for U of T graduates for the Greater Toronto area.

Heggeness and colleagues [16] used public data from the US census to build a comprehensive picture of career outcomes for PhDs in the biomedical sciences (http://www.sjscience.org/article?id=570). In 2004 there were 26,000 individuals under 40 working as biomedical scientists, mostly as trainees. By 2011 this number had increased to 36,000 with four out of five working outside academia in good agreement with our findings (18% of Life Sciences PhD graduates are employed as tenure-track professors with an additional 7% adjunct professors working in hospital-based research institutes). They highlight the “need to gather and communicate data about what careers past trainees have followed so that current trainees can benefit from this experience.”

An analysis of the 2010 Survey of Doctoral Recipients (SDR) found that over 60% of the STEM PhD holders who graduated from 1959 to 2010 are currently working in the United States are employed in non-academic careers mostly for private businesses mostly in R &D or in government [17]. Women and minorities were more likely to work in government or non-STEM fields than other groups performing work unassociated with R&D. They conclude “PhD students lack training in areas that may feature strongly in their career pursuits.”

It would be very useful to compare the employment outcome data obtained in the 10,000 PhDs project to similar studies carried out at universities in Canada, the USA and internationally. As mentioned in the Introduction, such comprehensive data is not readily available and, in some cases, different methodologies and survey instruments were used making comparisons difficult. It will perhaps fall onto national organizations such as the Canadian Association for Graduate Studies (CAGS) in Canada and Council of Graduate Schools (CGS) in the United States to coordinate PhD employment outcome activities in their constituent universities to produce robust and comparable datasets using common methodologies. The formation of the Coalition for Next Generation Life Sciences is an important step in this direction [11]. The 10,000 PhD Project provides a template for other universities to conduct similar studies without the use of expensive surveys that often have poor completion rates.

### Informing graduate education

The realization that the majority of PhD graduates do not assume tenure-track positions can inform and indeed transform graduate education. The apprenticeship model whereby professors essentially train their replacements is outmoded. In today’s dynamic economy and job market, academic supervisors need to embrace and indeed, celebrate the diversity of careers their PhDs graduates obtain, as many graduates move well beyond the comfortable confines of the academic world. A 2010 survey of 4109 PhD science students at 39 top US research universities found that most students, often with the encouragement of their advisors, initially aspire to a faculty position with a focus on research but over the course of their graduate studies this goal becomes less attractive, perhaps after experiencing the challenges of academic life first-hand or learning about other rewarding career paths [18].

Graduate students often find the transition from school to work difficult, highlighting the need to embed professional development within graduate programs, where students can develop their transferable skills and professional networks [19,20]. Skill gaps analyses in Canada [21] and the US [22] have highlighted the importance of equipping trainees with the skills (leadership, project management, communication, problem-solving, programming, financial and process improvement), etc. employers, especially those employers outside academia, are looking for.

A comprehensive Biomedical Research Workforce Working Group Report [23] released in 2012 recommended that “NIH should create a program to supplement training grant through competitive review to allow institutions to provide additional training and career development experiences to equip students for various career options and test ways to shorten the PhD training period.” The Report also concluded that the transition of PhDs into the biotech and pharmaceutical industries would be more effective if their training were better aligned with the skills required in these careers. One outcome of this Report is that NIH grantees that support graduate students for doctoral degrees and/or postdoctoral researchers should have Individual Development Plans (IDPs) for these individuals (http://myidp.sciencecareers.org/). The BEST Consortium [24], funded by NIH, is clearly aimed at implementing best practices to better prepare trainees for diverse career options.

The data from the 10,000 PhDs Project was made available to all Faculties and graduate units within U of T once the searches and data analysis was completed. Further information gleaned from departmental records on their alumni was used to locate an additional 3% of graduates and reduce the number of unknowns. To ensure transparency, this updated employment data of 88% of the graduates is publically-available in an easy to navigate dashboard format on the SGS web-site (http://www.sgs.utoronto.ca/about/Pages/10,000-PhDs-Project.aspx). Prospective PhD students can use the outcome data to imagine their career possibilities in different disciplines and current graduate students can make more informed career choices.

## Conclusion

The 10,000 PhDs Project has shown that PhD graduates from the University of Toronto continue to find employment in the post-secondary education (PSE) sector as professors, but increasingly are finding jobs in other sectors helping to drive an innovation economy. This should not be surprising given their highly-evolved communication, research and technical skills, strong work ethic, and ability to work independently and effectively in multi-disciplinary teams. These are the attributes among the knowledge generators, critical thinkers, innovators and problem solvers that the world needs today.

## Supporting information

S1 Methodology and Survey Form (PDF)
