# Supplementary material for: The 10,000 PhDs project at the University of Toronto: Using employment outcome data to inform graduate education
Source: PLoS One. 2019 Jan 16;14(1):e0209898. doi: 10.1371/journal.pone.0209898 (PMC6334897; doi:10.1371/journal.pone.0209898)
Supplement: S1 Methodology and Survey Form — (PDF) [file pone.0209898.s001.pdf]

Supporting Information

S1 Methodology and Survey Form

The survey form (see below) included 5 sections that required researchers to input respective information into the following sections and fields.

The first section to be filled out was the CURRENT EMPLOYMENT section, which was divided into Primary Employment and Secondary Employment to consider other potential positions graduates may currently hold. Both sections included the following input fields: position, start-date (YYYY-MM-DD), Employer, Employer Address, City, Province/State/Region, Country of Employment.

The second section to be filled out was the PREVIOUS EMPLOYMENT section, which included the input fields: position, start-date, end-date, Employer, Employer Location (City, Province/State/Region, Country). Researchers had the option to insert multiple PREVIOUS EMPLOYMENT sections to account for multiple PREVIOUS EMPLOYMENT positions held. For example many faculty members may have been employed previously as a post-doctoral fellow.

The third section to be filled out was the FIRST EMPLOYMENT AFTER GRADUATION, which included the same input fields from the PREVIOUS EMPLOYMENT section. If it were the case that the alumni currently occupied the same position since they first graduated, the researcher would have selected the option that read *same as current employment*.

The fourth section to be filled out was the EMPLOYMENT DURING GRADUATION and included the same input fields as PREVIOUS EMPLOYMENT. This section was intended to incorporate employment held during the student's PhD, whether the student worked full-time and completed their PhD part-time or held part-time employment and were full-time PhD students.

The fifth section to be filled out was the FURTHER EDUCATION section which included the following input fields: Position, Institution Name, Institution Location (City, Province/State/Region, Country), start-date, end-date. This section was intended any further education students pursued after their PhD (ex: medical school). See Information for Post-Doctoral Fellows and Further Education, under Data Categorization for 5 Main Employment Sectors/PSE.

The remaining 9 sections of the survey required researchers to select options from drop-down menus.

The first section, PhD FIELD OF STUDY, was selected from the Statistics Canada Classification of Instructional Programs (2011) in order to standardize degrees offered at U of T with census data.

The second section, ACTIVELY ENGAGED IN RESEARCH, included the following options: very much, somewhat, not at all, N/A. Once an option was selected, researchers would mark the type of research (ex: basic, clinical, applied, etc.) Selection was left for researcher discretion but was heavily influenced by the alumni's publication history and job-related responsibilities.

The third section, FIVE MAIN EMPLOYMENT SECTORS, was made in order to organize the various sectors of employment found within the Canadian economy. These main sectors include: Post-Secondary Education (PSE), Public, Charitable/Non-for-profit (NGO), Private, Individual. Once a main employment sector was selected, researchers would select the specific SECTOR (fourth section) the alumni was employed in. Both FIVE MAIN EMPLOYMENT SECTORS and sub-sectors referenced information from the PRIMARY CURRENT EMPLOYMENT section at the beginning of the survey.

The fifth section, TOP 3 ESSENTIAL SKILLS, was used to assess the top 3 necessary skills for the job. These were left to the researcher discretion.

The sixth section, DATA SOURCES UTILIZED FOR INFORMATION, allowed researchers to keep track of the sources used to obtain information about the alumnus.

The seventh section, ONLINE PRESENCE, MEDIA, OFFLINE, was used as a de facto reference list for websites used in the search strategy in the case of inputted information needing to be reviewed later.

The eighth and ninth sections, NOTABLE GRADUATE and COMMENTS, were used to highlight if the researcher considered the alumni to be a notable graduate with an interesting or unusual or high-level leadership position and insert their reasoning in the comment section. The comment section was also used to list any other comments about the alumni, search strategy, etc.

Search Strategy

Starting with the year of graduation, the full name and field of study, researchers conducted systematic searches on Google to determine the career trajectory of the 10,886 PhD graduates. The search strategy was 3-fold, each investigating different aspects of career information about a given alumnus.

Searches began (referred to as a primary search) by inputting the alumni's First name and Last name into the search bar with additional identifiers (e.g., PhD, University of Toronto; field of study, etc.) being added to filter results. If the alumni's current employment was found, the information was verified from another source. If verification was deemed successful, employment information was then inputted into the survey form. Previous employment history, once verified was inputted into the survey form however, researchers would proceed to the next stage of searching (referred to as a secondary search) to determine current employment information.

A secondary search required a search of the publication history of the alumnus to determine location/institution information as well as time spent at the institution by assessing author affiliation and publication dates, respectively. Google Scholar was the primary database used with these search attempts. If relevant information was found, the new information was then used to conduct a new primary search of the individual's current employment and as such governed by the same procedure mentioned in a primary search. The creation of a new primary search was used as data quality control measure to add additional sources that supported the finding. If this search was unsuccessful in determining current employment, researchers proceeded into a final tertiary search.

A tertiary search required a search of the alumni's supervisor or home department websites to retrieve any publically-available alumni information from these sources. Amongst STEM (Science, Technology, Engineering, Mathematics) fields, supervisors tended to have lab personnel pages with varying information about lab alumni. If relevant information was found in this attempt, the new information was then used to conduct a new primary search for more relevant information.

Unsuccessful attempts after tertiary searches were collected and distributed to respective departments to determine whether individual departments had information on alumni that was not accessible online. Departments would verify employment status individually and input career information into the database. If this attempt was deemed unsuccessful, the alumni was then considered to be not found and current employment was categorized as Unknown.

Reliability of Sources

The following is a list of the most commonly utilized internet data sources accessed by an initial Google search in order of their utility:

1. Google Scholar
2. University Directory
3. Website (work-place)
4. Linkedin
5. On-Line (publications, CVs)
6. Research Gate
7. Website (personal)
8. Academia.edu
9. Academic Directories/ Databases
10. Twitter
11. Other
12. ZoomInfo
13. Google+
14. Facebook
15. Beyond.com
16. EFactor

Certain websites were deemed more reliable in terms of information provided and were determined suitable through standardized practice and researcher discretion. For example, faculty web-site pages, employee directories and LinkedIn were considered very reliable whereas Rate My Professor, ZoomInfo were not as reliable. The reliability of blogs and web-publications were subject to date of publication but could be considered useful for both further search queries and employment categorization. Additionally, if information was found about the alumni being registered to a Professional Society (College of Physicians and Surgeons, Law Society of Upper Canada, Professional Engineers, College of Psychologists, etc.), a search for the alumni in those respective databases was conducted.

General Tips

1. Ratemyprof is a good resource to assess dates and time spent at a specific institution but should not be considered as a source for employment type
   - Institution information can be used as an identifier for a Primary Source
   -
2. Public databases (ex: Ontario Doctor Directory, Psychologists Today) are databases that pull information from official licensed professional memberships directories (ex: CPSO, CPO), as the official directories are not accessible through a Google search. Therefore, if a researcher encountered the alumni in a public directory, they would conduct a name search in the respective directory to obtain member information
   - The directory for Professional Engineers is another great resource for all licensed Engineers (both in the PUBLIC and PRIVATE sectors) which provides employment position, employer and

location data, however it does require that the alumni have a Bachelor's of Engineering degree.

- It is important to note that while most PhD Engineers have an engineering undergraduate background, this is not always the case.

Data Categorization for 5 Main Employment Sectors

The following section provides definitions of sub-sectors, under the FIVE MAIN EMPLOYMENT SECTOR. Additionally, we provide general tips, assumptions and reasoning for the specific decisions on how alumni were categorized.

Post-Secondary Education (PSE)

- **Definitions:**
  - **University Professor (Assistant/Associate/Full Professor, full-time, tenure-track, research and teaching, administration)**: Individuals are based at and are paid by the University in tenure-stream positions. They typically conduct research pertinent to their field of study, supervise graduate students and perform administrative and teaching duties. In most Canadian universities tenure accompanies promotion to Associate Professor.
  - **University Professor/Lecturer (may also be considered as a University Professor, Teaching Stream)**: Similar to the above, however these individuals do not conduct field-of-study research but rather pedagogical research related to their field of study. Their main duties surround teaching and administration. Note that is some countries like the UK, Australia and New Zealand the term Lecturer or Fellow is often used for these positions.
  - **University Professor (full-time, status-only, adjunct, affiliated research institute)**: Individuals have the same rank of a tenured/track professors but are based at and are paid by their home institutions (ex: research institutions, hospitals, etc.) that are affiliated with a university. They typically supervise graduate students and post-doctoral fellow, hold research grants and publish in peer-reviewed journals. A useful identifier for this group can be found by the alumni's email domain.
  - **University Professor (part-time, research and/or teaching):** Individuals who tend to hold an adjunct professor position and conduct research but have different primary employment outside of the PSE sector. For example, some may be employed in the private sector. This position is different from a **Research Associate/Assistant/Contract** whose main responsibilities only require field of study research and are professionally employed at a University or research institute (see breakdown of Research Associate and Post-Doctoral Fellow below)
  - **Part-time/Contract/Sessional Lecturers:** Individuals do not conduct research and primarily lecture on a contractual basis. This category is most common in the Humanities.
  - **College Lecturer (Full Time/Part-time):** Individuals who work at a registered college. They can sometimes conduct research but primarily teach. Part-time and full-time designations refer to

individual's employment status and are separated in the original survey form. Colleges is Canada are typically non-degree granting, however in the United States College most commonly refer to small universities focused on undergraduate education.
  - **University or College Administration/Service Provider:** Individuals solely preform administrative duties or services for the post-secondary institution they are employed at.
  - **Post-Doctoral Fellow:** Individuals conduct research and are considered as pre-faculty trainees. They however are employees of the institutions they are based at and follow a specific categorization procedure mentioned below.
  - **Further Education:** These are individuals who choose to pursue further education (Medical School, Law School, MBA, etc.) after their PhD and are currently still in school.

- **Information about Post-Doctoral Fellows, Research Associates and Further Education:**
  - Information that an alumnus is currently a Post-Doctoral Fellow but if information about institution and date information is lacking, an employment history can be created based from the alumni's publication history. It is important to note that the accuracy rate is much higher for alumni in publication heavy fields of study like Life and Physical Sciences.
  - Post-Doctoral Fellows are often considered employees of their university or research institution but are also considered trainees (i.e. pursuing a form of further education).
    - Therefore, if the alumni is pursuing a Post-Doctoral Fellowship at time of categorization, the information will be treated as a form of employment to be filled out in the CURRENT EMPLOYMENT section (whereby the position is POST-DOCTORAL FELLOW and the employer is INSTITUTION NAME).
    - If the alumni conducted previous post-doctoral fellowships prior to their current one, these were categorized under the corresponding PREVIOUS EMPLOYMENT sections.
    - The similar information was then copied into the FURTHER EDUCATION section into the respective fields (position, institution name…)
    - The similar approach was conducted for individuals who pursued further education after their PhD, which followed this notation

Position = [respective field of study] student, employer = institution name

  - If a Post-Doctoral Fellowship is being conducted at a private institution such as an industrial fellow with a company,

categorization follows the same procedure as the above, however the company information will be listed as the INSTITUTION NAME and EMPLOYER fields respectively.

- The alumni will still be categorized as a **Post-Doctoral Fellow** (PSE sub-sector) within the 5 main Employment Sectors section of the survey.
- The same should be done if a Post-Doctoral Fellowship is being conducted at an NGO/ Non-for-Profit organization carrying out research.

o Research Associates are permanent full-time researchers at universities and research institutes and are different from Post-Doctoral Fellows who also conduct research full-time. The differentiating factor is such that a Post-Doctoral Fellowship is a training program usually initiated after the end of one's PhD and tend to last a maximum of 5 years.

- Post-Doctoral Fellows can become Research Associates however the inverse is rarely seen.
- Post-Doctoral Fellows and Post-Doctoral Research Associates are the same position but have different funding models. Post-Doctoral Fellows are funded primarily through grants whereas Post-Doctoral Research Associates are paid by the institution.

- **General Information**
    - If an individual is a tenured/tenure-track professor and preforms administrative duties (ex: are a department chair/dean/university president, etc.), they were categorized under the **University Professor (Assistant/Associate/Full Professor…)** sub-sector.
    - Institutional faculty and staff directories are a great resource for verifying the information of the alumni's employment within the PSE sector. However, one should also check departmental directories if the institution-wide search is unsuccessful.
    - If a researcher was not able to distinguish between a college and a university (many undergraduate-only universities in the US use the identifier college in their name), the following definitions were used:
        - Does the institution award 4-year degrees? If so, it is classified as a university.
        - Does the institution offer graduate programs? If so, it is classified as a university.
        - Does the institution offer 2-year diplomas? If so, it is a college.
        - Is the institution a College-University partnership for specific programs?
            - If the alumni is based at the university, they are a **University Lecturer**
            - If the alumni is based at the college, they are a

College Lecturer

- o Private, Public, For-Profit and Non-for-Profit universities were all considered as universities during categorizations.
  - ▪ The same was done for respective colleges.
- o Health Providers (Physicians, Nurses, Psychologists, etc.) who work at the institution are categorized under **Administrators/Service Providers**.
- o Course Instructors/Directors/Coordinators, Curriculum Developers were all considered to be different forms of forms of **Part-time/Contract/Sessional Lecturers** as these are critical components in curriculum development.
- o Established Institutions such as Oxford, Cambridge and Harvard University as well as others may tend to use different designations for faculty. Here are some of the following we encountered:
  - ▪ Readers = Associate Professor
  - ▪ Lecturers = usually considered as Assistant Professors, if the institution followed the UK Educational System, but more information is needed
  - ▪ Preceptor = Sessional or Contractual Lecturer or Course Administrator

Public Sector

- **Definitions:**
  - o **Government**: Individuals work for various levels of government.
    - ▪ Crown Corporation employees were categorized in the PRIVATE SECTOR (see explanation within PRIVATE SECTOR).
  - o **Hospital**: Individuals work at a hospital, regardless of employment type (healthcare professional, hospital administration, etc.). Researchers who are not affiliated with a university but are based at a hospital are categorized under this sub-sector.
  - o **Health-Related (non-hospital based)**: A healthcare professional who does not work at a hospital. Health administrators not at a hospital were also included under this category.
    - ▪ An example of this type of individual would be a physician who runs their own health clinic.
  - o **Education:** Individuals work within the public schooling system, regardless of employment type (teachers, administrators, etc.)
  - o **Broader Public Sector:** Individuals are considered as public sector employees but do not fit within the categories mentioned above

General Information

- o Government Employee databases/directories are good resources to find employee status and information (ex: Government Electronic

Directory Services for federal Employees and INFO-GO for Ontario provincial employees).
    - Other Useful Professional Directories can include the College of Physicians and Surgeons of Ontario (CPSO), College of Psychologists (CPO).
    - The Ontario College of Teachers (OCT) can provide information on whether a licensed teacher (in Ontario) is in good standing but will not tell you if and where the individual is currently working as a teacher. Another source (we-page, publication, etc.) was needed in addition to the designation of *Good Standing* in the OCT.
    - The categorization for Medical Residents follows the similar format as Post-Doctoral Fellows (mentioned above) with respect to the CURRENT/PREVIOUS EMPLOYMENT/FURTHER EDUCATION, however they were categorized under the **Hospital** or **Health-Related** sub-sectors, within the 5 main Employment Sectors section of the survey.

Charitable / Non-for-Profit / NGO Sector

- **Definitions about sub-sectors were relatively straight-forward and left to researcher discretion to decide fit.**
- **General Information**
    - Non-for-Profit research institutions (ex: think-tanks) that are not affiliated with hospitals or universities but have individuals who conduct research like those conducted at a university are categorized under this sector
        - Administrators who work for these research institutes are also categorized in this sector
    - The World Bank, United Nations, World Health Organization employees are categorized under this sector can fit under the respective sub-sectors: **Research and Policy**, **Human Rights and Dignity** and **Health-Related**.
    - Public Museums employees (Researchers not affiliated with a university and Administration) are categorized under **Arts and Culture**

Private Sector

- **Definitions about sub-sectors were relatively straight-forward and left to researcher discretion to decide fit.**

- **General Information**
    - Crown Corporation employees were categorized as Private Sector Employees.

    - By definition, Crown Corporations are corporations that are regulated by the government and provide public goods/trusts like transportation and energy (ex: TTC and Ontario Power Generation).
    - While partly subsidized by the government, individuals must pay to use these services, which is the reason why employees are categorized in the PRIVATE SECTOR and under respective sub-sector.
  - If individuals work for a private company or corporation as Research Scientists and conduct similar types of research as they would at a university, they were still categorized in the PRIVATE SECTOR and under the respective sub-sector.
  - Health Professionals that are not funded publically and require individuals to pay for the service or have insurance (ex: Dentistry, Psychologists, Psychiatrist, Therapists, Rehabilitation and Physiotherapy, etc.) were categorized in the PRIVATE SECTOR and under the sub-sector OTHER, which was to be specified.
    - *The specification format is as follows: Health (insert specialty).*
    - This categorization was standardized whether the alumni operated their own private clinic or worked in conjunction with other specialists.
  - If an individual works as a teacher or administrator for a private school (K-12), they were categorized in the PRIVATE SECTOR and under the sub-sector OTHER, which was to be specified.
    - *The specification format is as follows: Private Education*

Individual Sector

- **Definitions:**
  - **Independent Business:** The individual owns and operates a business with a physical address.
  - **Self-Employed:** The Individual owns and operates their own business but do not have a physical address for their business (ex: E-commerce).
  - **Consultant**: The individual strictly provides consulting services by themselves.
    - If the individual is a consultant for a company, they were categorized in the PRIVATE SECTOR, under the **Management and Administration** sub-sector.
  - **Family Care**: The individual is not in traditional job market and provides care to family members.
  - **Unemployed**: The individual is unemployed.
  - **Out of Labour Market (on the survey form, this category reads as Retired)**: The individual is retired or has since passed away.

- **General Information:**
    - If the individual is retired, categorizing the individual for current employment is as follows: *Position = Retired, Start Date = Date of Retirement, Employer: Retired, Location: input location from most recent previous employment)*
    - If the individual is deceased, *replace the position and employer fields with Deceased* and note that they are deceased in the comment section.

Software Used and Data Analysis

The survey form was hosted on a secured Microsoft SharePoint file on administration servers. Once completed, the results from the survey were exported to a secured Microsoft Excel file again on administration servers to later be analyzed. Figures and percentages were generated using pivot tables of the exported data and are represented in the *Results* section below.

Research Protocol

The research protocol for the PhD project was reviewed by the Research Oversight and Compliance Office at the University of Toronto. Because the project would only be accessing publically-available data and no individuals would be contacted as part of this research project, the Office confirmed that Research Ethics Board approval would not be required.

SGS 10,000 PhDs Employment Survey

Personal Data 1 SGS (read-only)

Record Number
(Student Number)
Year of Graduation
Last Name
First Name(s)/Alternate First Name/Middle Name or Initial

Gender:

Status In Canada:

Country of Citizenship:

Division:

Graduate Unit:

Program Name:

Attendance Type:

Supervisor:

Co-Supervisor:

Thesis Title:

**SGS Divisional Structure**		DIVISION CODE
Division I	Humanities	HUMGS
Division II	Social Sciences	SSCGS
Division III	Physical Sciences	PHSGS
Division IV	Life Sciences	LFSGS

** START OF DATA COLLECTION **

Personal Data 2

Public Contact Information

Email:

LinkedIn:

Other:

Employment Career Path

Current Employment Position (Primary)

Position:

Start Date (DD/MM/YYYY):

Employer:

Location Address:

Location City:

Location Province/State/Region:

Location Country:

Current Employment Position (Secondary)

Position:

Start Date (DD/MM/YYYY):

Employer:

Location Address:

Location City:

Location Province/State/Region:

Location Country:

Previous Employment Positions

Position:

Start Date (DD/MM/YYYY):

End Date (DD/MM/YYYY):

Employer:

Location:

First Employment Position After Graduation

Same as Current Position

Position:	
Start Date (DD/MM/YYYY):	
End Date (DD/MM/YYYY):	
Employer:	
Location:	
Other:	

Employment Position During Graduate School

Position:	
Start Date (DD/MM/YYYY):	
End Date (DD/MM/YYYY):	
Employer:	
Location:	
Other:	

Further Education After Graduation (Post-doctoral Fellow, Professional School, etc.)

Program (Medical School, etc.) or Position (PDF)	
Institution:	
Location:	
Start Date (DD/MM/YYYY):	
End Date (DD/MM/YYYY):	
Other:	

PhD Field of Study

- ○ Agriculture/Forestry
- ○ Architecture
- ○ Computing/Information Sciences
- ○ Education
- ○ Engineering and Applied Sciences
- ○ Humanities
- ○ Law

- Life Sciences
- Management/Business
- Medical/Health Sciences
- Music
- Physical Sciences
- Social Sciences
- Social Work
- Other (specify)

Active in Research/Scholarly/Teaching Activities Related to Field of Study/Discipline

Regularity of Activity:

- Very much so
- Somewhat
- Not at all
- Unknown

Type of Activity:

- Basic research
- Applied research
- Clinical research
- Humanities research
- Social sciences research
- Teaching
- Professional training
- Research administration
- Consulting
- Other (specify)
- N/A

Five Main Employment Sectors

- 1: Post-Secondary Education (PSE) University Professor/ Lecturer/ Administration/Further Education

- 2: Public Sector (Government/Hospital/Education/Broader Public)
- 3: Charitable Sector (NGO/Not-for-profit/Community)
- 4: Private Sector (Industry)
- 5: Individual Sector (Consulting)
- Other
- Unknown

Sectors

Sector 1: Post-Secondary Education (PSE)

- University Professor (Assistant/Associate/Full Professor, full-time, tenure-track, research and teaching, administration)
- University Professor/Lecturer (full-time teaching, administration)
- University Professor (full-time, status-only, adjunct, affiliated research institute)
- University Professor (part-time, research and/or teaching)
- University Lecturer (part-time/sessional/contract teaching)
- College (non-degree) Lecturer (full-time teaching)
- College (non-degree) Lecturer (part-time/sessional/contract teaching
- University or College Administration/Service Provider (full-time)
- University or College Administration/Service Provider (part-time)
- Research Associate/Assistant/Contract (full-time)
- Other (specify)

Further Education

- Post-doctoral Fellow
- Medical School
- Dental School
- Pharmacy School
- Nursing
- Applied Health
- Veterinary School
- Business School

- Law School
- Teacher’s College
- Community College/Technical Training
- Other (specify)

Non Post-Secondary Education Sectors

Sector 2: Public Sector

- Government
- Hospital
- Health-services (non-hospital based)
- Education
- Broader Public Sector
- Other (specify)

Sector 3: Charitable Sector

- Health-related
- Environment
- Community Development
- Arts and Culture
- Education
- Social Services
- Research and Public Policy
- Human Rights and Dignity
- Religious
- Animal Welfare
- Sports and Entertainment
- International/ NGO

Sector 4: Private Sector

- Information Technology, Internet, Social Media (Google, Microsoft, Facebook)
- Banking, Finance, Investment
- Industry, Research and Development
- Biotechnology/Pharmaceuticals
- Engineering/Computing Technology
- Law and Legal
- Management and Administration
- Media, Publishing
- Marketing/Advertising
- Event Planning
- Public/Government Relations
- Sales and Service
- Arts, Culture
- Sport, Recreation
- Environment, Agriculture
- Trades, Transport, Equipment Operation, Manufacturing
- Other (Specify)

Sector 5: Individual Sector

- Independent Business
- Self-employed
- Consulting
- Family Care
- Unemployed
- Retired
- Other (specify)

Multiple-Sectors (PSE + another)

Essential Skills and Core Competencies (Pick Top 3 from LinkedIn)

- ☐ Communication
- ☐ Information Gathering And Data Analysis
- ☐ Innovation/Entrepreneurship
- ☐ Leadership/Mentoring
- ☐ Project Management
- ☐ Research Related Skills
- ☐ Strategic Thinking
- ☐ Teaching And Knowledge Translation
- ☐ Other (specify)

Data Sources Utilized for Information

- ☐ Academia.edu
- ☐ Academic Directories/Databases
- ☐ Beyond.com
- ☐ EFactor
- ☐ Facebook
- ☐ Google
- ☐ Google+
- ☐ LinkedIn
- ☐ On-line (Publications, CVs)
- ☐ Research Gate
- ☐ Twitter
- ☐ University Directory
- ☐ Website (Work-place)
- ☐ Website (Personal)
- ☐ Zoom Info
- ☐ Other (specify):

Online Presence, Media, Offline

Web Site URLs:<br>(one per line)	
Twitter Handle:	
Podcasts<br>(YouTube, Ted Talks…):<br>(one per line)	

News Paper, Magazine:<br>(one per line)
Previous Talks:<br>(one per line)

Notable Graduates

Name:
Position:

Comments

